# Aqueous Metabolite Trends for the Progression of Nonalcoholic Fatty Liver Disease in Female Bariatric Surgery Patients by Targeted ^1^H-NMR Metabolomics

**DOI:** 10.3390/metabo11110737

**Published:** 2021-10-27

**Authors:** Emma J. Robinson, Matthew C. Taddeo, Xin Chu, Weixing Shi, Craig Wood, Christopher Still, Virginia G. Rovnyak, David Rovnyak

**Affiliations:** 1 Department of Chemistry, Bucknell University, 1 Dent Drive, Lewisburg, PA 17837, USA; ejr026@bucknell.edu (E.J.R.); mct013@bucknell.edu (M.C.T.); 2 The Obesity Institute, Geisinger, Danville, PA 17822, USA; xchu@geisinger.edu (X.C.); wwshi@geisinger.edu (W.S.); cwood@geisinger.edu (C.W.); cstill@geisinger.edu (C.S.); 3 University of Virginia School of Nursing, Charlottesville, VA 22908, USA; vgr4e@virginia.edu

**Keywords:** nonalcoholic fatty liver disease, NAFLD, obesity, nuclear magnetic resonance, NMR, metabolomics, bariatric surgery

## Abstract

Determining biomarkers and better characterizing the biochemical progression of nonalcoholic fatty liver disease (NAFLD) remains a clinical challenge. A targeted ^1^H-NMR study of serum, combined with clinical variables, detected and localized biomarkers to stages of NAFLD in morbidly obese females. Pre-surgery serum samples from 100 middle-aged, morbidly obese female subjects, grouped on gold-standard liver wedge biopsies (non-NAFLD; steatosis; and fibrosis) were collected, extracted, and analyzed in aqueous (D_2_O) buffer (^1^H, 600 MHz). Profiled concentrations were subjected to exploratory statistical analysis. Metabolites varying significantly between the non-NAFLD and steatosis groups included the ketone bodies 3-hydroxybutyrate (↓; *p* = 0.035) and acetone (↓; *p* = 0.012), and also alanine (↑; *p* = 0.004) and a putative pyruvate signal (↑; *p* = 0.003). In contrast, the steatosis and fibrosis groups were characterized by 2-hydroxyisovalerate (↑; *p* = 0.023), betaine (↓; *p* = 0.008), hypoxanthine (↓; *p* = 0.003), taurine (↓; *p* = 0.001), 2-hydroxybutyrate (↑; *p* = 0.045), 3-hydroxyisobutyrate (↑; *p* = 0.046), and increasing medium chain fatty acids. Exploratory classification models with and without clinical variables exhibited overall success rates ca. 75–85%. In the study conditions, inhibition of fatty acid oxidation and disruption of the hepatic urea cycle are supported as early features of NAFLD that continue in fibrosis. In fibrosis, markers support inflammation, hepatocyte damage, and decreased liver function. Complementarity of NMR concentrations and clinical information in classification models is shown. A broader hypothesis that standard-of-care sera can yield metabolomic information is supported.

## 1. Introduction

Nonalcoholic fatty liver disease (NAFLD) is associated with metabolic syndrome (MetS) and is characterized as an epidemic [1,2]. The growing burden on healthcare providers, the increased indication for liver transplants, and an increasing NAFLD-related death rate are serious socioeconomic impacts of this epidemic [3,4,5].

The understanding of MetS and its associated conditions is being pursued through the study of metabolic dysregulation. For example, elevated levels of the most abundant blood proteins report on MetS [6,7], while the risk and progression of Type 2 diabetes (T2D), cardiovascular disease (CVD), and insulin resistance (IR) have been associated with elevated branched chain amino acids (BCAAs; isoleucine, leucine and valine; ILV) [8]. While T2D is perhaps the most extensively investigated disease by metabolomics, NAFLD is now being associated with changing metabolite levels [9,10,11,12,13,14,15,16]. Associating specific metabolites with mileposts in the spectrum progression of NAFLD remains a challenge, particularly due to comorbidities, such as T2D, which often progress in concert with NAFLD, and to the participation of the gut microbiome in metabolism [17]. Yet if the biochemical pathology of the progression of NAFLD can be unraveled, it may enable early detection of liver disease or help identify subjects at risk to advance beyond steatosis.

Mass spectrometry has been applied widely to measure metabolite markers of NAFLD, while the use of NMR methodology is more limited but growing [10,13,18,19,20]. These methods are generally complementary, where both can provide quantitative, targeted analysis of a useful subset of metabolites. This work employed a curated (non-high-throughput) NMR approach with the aim of further extending the precision and limits of NMR quantitation to enhance the ability to discern metabolite trends relative to biopsy-proven NAFLD groups. As the potential to identify small molecule biomarkers of NAFLD is growing, this work also tested the ability to identify biomarkers in retrospective cohorts consisting of biobanked, standard-of-care samples, where there is a need to determine the suitability of such resources for metabolomic information [7]. Collected over non-consecutive time spans, with less control of diet, fasting, interventions, supplements, medication, compliance, and phlebotomy conditions, for example, such sera are expected to possess more variation than would occur in prospective clinical trials in which such variables are often controlled. Conducting biomarker validation and discovery in more variable patient groups tests the metabolic information content of biobanked sera [7], but also tests the potential for such biomarkers to have diagnostic potential in a broader population.

As noted, a challenge is to distinguish markers solely for the progression of NAFLD in the presence of metabolic trends of comorbidities that are commonly associated with NAFLD. Obesity, CVD, IR, and T2D are associated with trends in some of the most abundant serum metabolites, including glucose and branched chain amino acids (BCAAs). Increasing BCAA in NAFLD could be caused by parallel progression of IR and T2D with NAFLD, but whether altered liver states contribute additionally to BCAA changes is difficult to discern. Furthermore, given differences in the prevalence, progression, and pathology of NAFLD between male and female subjects [21], identifying sex-specific metabolite trends will contribute to the understanding of NAFLD progression. Understanding sex-specific trends in metabolism is a growing need in the study of metabolic disorders [22].

In this work, NMR-derived markers are reported for steatosis and fibrosis relative to a non-NAFLD liver in groups selected from a convenience sample of a population of morbidly obese female bariatric surgery patients. A low influence of T2D and obesity between the groups in this study is noted and suggests that the observed markers in this study are sensitive to changing liver health.

## 2. Results

All subjects had very high body mass index (BMI) (Table 1), and their sera were often visually lipemic and likely to contain high lipid content, even if not milky. Obesity can strongly affect metabolism and metabolite variation [23]. In this sample, however, BMI was consistent across the three subgroups studied.

Although acetonitrile and water are miscible in all proportions, an interesting result connected to this work was the discovery of a liquid–liquid phase separation during the extraction of the majority of the samples [24], where high lipids likely constitute a third component and cause the observed phase separation. To reproducibly and reliably obtain aqueous metabolites, a slightly modified extraction protocol was implemented for the sera used in this study that includes a brief room-temperature step to break the liquid–liquid phase separation; this simple modification preserves the removal of proteins and may be of interest in metabolomics of obesity-related diseases [24].

### 2.1. Patient Characteristics and NMR-Derived Metabolite Levels

This work is a retrospective study of biobanked sera from 100 high BMI female subjects consented during a preoperative standard-of-care draw for bariatric surgery. The characteristics of the three groups are summarized in Table 1. Subjects were classified on assessment of liver sections, which is the gold standard for determining NAFLD progression. The progression of NAFLD in the groups in Table 1 is also illustrated by insulin, alanine aminotransferase (ALT), aspartate aminotransferase (AST), and waist circumference, which were observed to vary significantly across the groups and have known associations with NAFLD [26,27,28].

A high BMI control group controlled the influence of obesity on metabolite trends [23]. The proportion of T2D diagnoses did not change across the groups in Table 1. Further, clinical markers associated with T2D did not change significantly across the groups either. Specifically, HDL and LDL levels were unchanged while triglyceride levels showed weak evidence for potentially changing (*p* = 0.074). As noted earlier, Metformin use was allowed and expected in T2D subjects, and others as well. Although the effect of Metformin use on the aqueous metabolome is not resolved, it may moderately stabilize some BCAA/AAA T2D biomarkers [29].

Importantly, markers often associated with T2D can also be affected by NAFLD progression. Notably, Hba1c and insulin levels are incorporated into diagnostic models of NAFLD [27]. Given the evidence that T2D did not change across the groups, the Hba1c and insulin levels are more likely to report on NAFLD progression in this work.

A summary of metabolite means (^1^H NMR, 600 MHz) and ANOVA (analysis of variance) results is given in Table 2, where a small number of significant metabolites are identified. One-way ANOVA analysis indicates seven metabolites that vary significantly across all three groups (*p* < 0.05), with one additional metabolite that is weakly suggested as significant (propylene glycol, *p* = 0.050). The significant metabolites in Table 2 generally show good agreement with the results of related studies and will be discussed in more detail below. Alanine has been noted previously as a potential marker but is one of the most significant metabolites in this work, while 2-hydroxyisovalerate has recently been reported with NAFLD progression [10]. The metabolites that change between the steatosis and non-NAFLD groups are distinct from the metabolites that distinguish the fibrosis and steatosis groups (Table 3).

Principal component analysis (PCA) was uninformative (not shown), confirming that intra-group variation exceeded inter-group variation. Such variation was expected, as retrospective standard-of-care sera of bariatric surgery patients are not subject to the same controls as a prospective clinical research setting. The biomarkers summarized in Table 2 and Table 3 confirm that meaningful metabolites can be detected as a function of NAFLD progression despite high intra-group variation. Consistent also with the substantive intra-group variation, no metabolites in this study exhibited *p* < 0.001 in either ANOVA tests across all groups or *t*-tests between groups. Finally, cluster analysis by PLSDA was conducted (Figure S1), which shows that favorable separation between different groups is obtained; although PLSDA can be prone to overfitting, variables associated with PLSDA clustering have substantial agreement with significantly varying means in Table 2 and Table 3.

### 2.2. Fatty Acid Oxidation: Steatosis vs. Fibrosis

**Figure 1:**
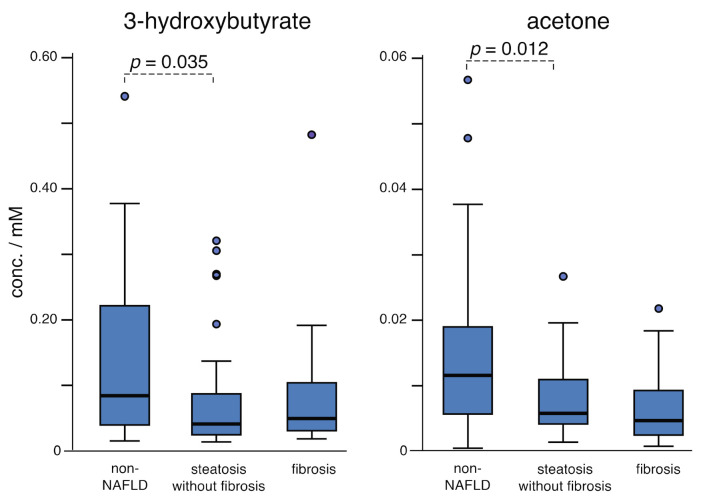
Boxplots illustrate decreasing ketone bodies in the steatosis group that are also depressed in the fibrosis group. Acetone may be influenced by the extraction, but its trend here is similar to that of 3-hydroxybutyrate, where each decreased by about two-fold across the groups. Acetoacetate is unreliable in these data but is explored in Figure S2 (Supporting Information). Multiply concentrations by 2 for the serum levels.

Inhibited hepatic fatty acid oxidation (FAO) is a part of the complex molecular pathology of NAFLD [30,31] and is evidenced in this study through the accumulation of triglycerides, weakly suggested in the ANOVA analysis (*p* = 0.074), and significant when comparing the non-NAFLD and fibrosis groups (*p* = 0.029). The data further show that the ketone bodies 3-hydroxybutyrate (3HB) and acetone decrease, particularly between the non-NAFLD and steatosis groups (Figure 1, Table 3).

Whereas 3HB can be assigned unambiguously and profiled in the ^1^H-NMR data, there are some experimental challenges for characterizing the remaining ketone bodies, acetone and acetoacetate. Acetone is assigned from a single NMR line and was supported by spiking several samples with dilute acetone solutions. Although vacuum drying has been associated with a loss of acetone [32], the relatively weak vacuum (1 torr) employed in this work may be protective, or possibly the high lipid content observed in these samples may also help sequester acetone. Finally, profiling of acetoacetate is adversely affected by the presence of stronger overlapping signals from more concentrated metabolites (see Supporting Information, Figure S2). Normally such confounding signals would exclude an underlying metabolite from consideration; however, we elected to profile acetoacetate due to its potential importance, with the understanding that the significance determined for acetoacetate may be unreliable. Decreasing acetoacetate has been reported in NAFLD, and it could be speculated that a decrease in the steatosis group is weakly suggested in this work (Figure S2), but this work does not resolve the significance of acetoacetate. Nevertheless, the 3HB and acetone data are sufficient to demonstrate the early decrease and sustained depressed levels of ketone bodies and show that a disruption of fatty acid oxidation is detectable at steatosis in this study (Figure 1).

The medium chain dicarboxylic fatty acids pimelate, azelate, suberate, and sebacate are detected in 1D-^1^H-NMR spectroscopy; however, due to their chemical similarity they involve overlapping signals in NMR spectra. In the conditions in this work (600 MHz, 14.1), suberate is expected to be confounded primarily with pimelate, while azelate is expected to be confounded primarily with sebacate, and larger profiling errors and standard deviations are expected for these cases. Despite these factors, accumulation of these medium chain fatty acids in the fibrosis groups is seen. Specifically, the suberate (pimelate) concentration increases significantly between the steatosis and fibrosis groups (Table 3), while the azelate (sebacate) concentration weakly suggests an increase between the steatosis and fibrosis groups as well (*t*-test; *p* = 0.057, Figure S3). We refrain from interpreting these trends specifically among these four dicarboxylic acids, but rather observe that the accumulation of medium chain fatty acids is demonstrated in these data and supports MCAD (medium chain acyl-CoA dehydrogenase) deficiency or inhibition in fibrosis, which could be a genetic deficiency of the MCAD enzyme or a dysregulation of upstream species such as malonyl CoA or PPARα (peroxisome proliferator-activated receptor).

These results portray a multi-step disruption of fatty acid oxidation that spans the entire progression of NAFLD. Reduced ketone bodies are localized to steatosis and suggest a milder disruption of ketogenesis at this stage. In contrast, accumulation of triglycerides and medium chain fatty acids, likely reporters of broader accumulation of fatty acids, are localized to fibrosis and signal an increase in the severity of FAO inhibition.

### 2.3. Comparing Steatosis and Fibrosis Groups

**Figure 2:**
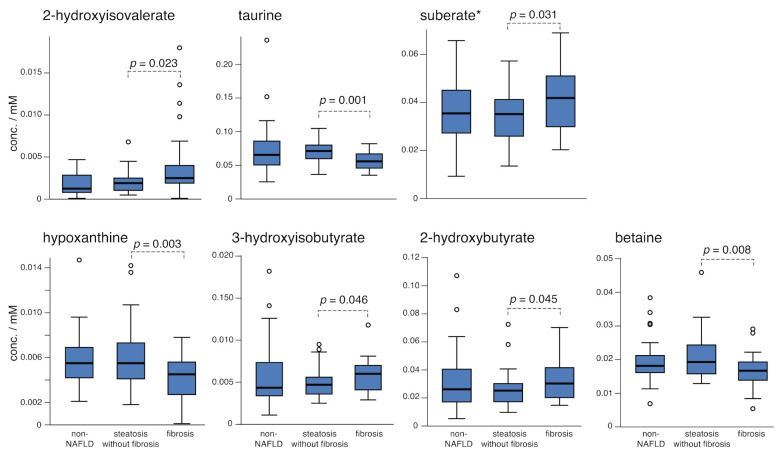
Boxplots of metabolite concentrations for which means changed significantly in this study between the steatosis and fibrosis groups (the non-NAFLD liver group is included for comparison). * Suberate profiling is confounded with another medium chain fatty acid pimelate (see additional medium chain fatty acids in Figure S3). See also Figure S4, where a weak accumulation of propylene glycol is suggested (*p* = 0.050). Multiply concentrations by 2 for the serum levels.

The metabolites that vary between fibrosis and steatosis are distinct from the metabolites that change between the steatosis and non-NAFLD liver groups (Table 3). A related observation that distinct metabolites characterize NASH progression has been observed [10]. While NAFLD is reasonably viewed as a spectrum condition, the distinct metabolite panel reporting on fibrosis speaks to the progression to fibrosis as a discretized step. Boxplots of the seven metabolites that are significantly altered between the fibrosis and steatosis groups are shown in Figure 2. The data for propylene glycol, which is weakly suggested as significant in fibrosis (*t*-test, *p* = 0.050), are illustrated in the Supporting Information (Figure S4). As an exogenous compound, significant variation of propylene glycol may be expected, and any trends in its levels could be more accurately characterized in controlled, prospective clinical tests. The weak accumulation of exogenous propylene glycol suggests a decrease in liver function to break down exogenous compounds, and its increase in liver tissue in NASH has been reported [18].

Disruption of taurine levels in fibrosis can indicate inhibited bile acid synthesis (e.g., tauro-conjugated bile acids), consistent with the understanding of loss of liver function in fibrosis. Decreasing serum betaine has been associated with progression of NAFLD, particularly with advanced stages [11,33], but detectable changes may occur as early as steatosis relative to a non-NAFLD liver. A decrease in betaine of about 20% was observed here in the fibrosis group relative to steatosis (Figure 2). Betaine is further suggested as decreasing with fibrosis progression from F1 to F2–3 stages (*p* = 0.058, Figure S5 in Supporting Information), suggesting that advanced fibrosis particularly influences the decreasing betaine levels observed here. Whereas serum betaine decreases with NAFLD progression, increasing betaine in liver tissue was observed in NASH, indicating betaine upregulation in the liver may be a protective response [18].

Among the more significant markers in this study, 2-hydroxyisovaleric acid has not been as widely associated with NAFLD but was noted recently in an MS study as a feature of fibrosis relative to a fatty liver [10] and is independently validated here by NMR methods. Further, 2-hydroxyisovalerate was associated with liver fibrosis in HCV patients [34]. Trends in 2-hydroxyisovalerate have been observed in diverse conditions and may be a general reporter of internal inflammation, which would be consistent with observing its most significant change here in the fibrosis group.

### 2.4. Branched Chain Amino Acids (BCAA) and Aromatic Amino Acids (AAA)

Levels of BCAA (isoleucine, leucine, valine) and AAA (phenylalanine, tyrosine, tryptophan) were not significantly changed with NAFLD progression between the three broad groups (non-NAFLD; steatosis; fibrosis; Table 1), but they did decrease between stage 1 (*n* = 16) versus stages 2 and 3 (*n* = 13) fibrosis levels, as presented in the next section.

Considering the broader groups first (non-NAFLD; steatosis; fibrosis), analyses within the T2D and non-T2D sub-groups, respectively, in this work also showed no significant trends in BCAA or AAA levels (not shown). Yet several studies of NAFLD have implicated branched-chain and aromatic amino acids [12,13,14,18], with perhaps more emphasis on BCAA trends. It is well known that changing BCAA and AAA levels are associated with increasing insulin resistance (IR) and T2D risk and progression. It is challenging to discern if the serum BCAA changes observed in NAFLD progression are due to hepatic damage itself; to confounding T2D risk or progression; or to broader metabolic outcomes of increasing BMI, insulin resistance, and waist. In this study, both T2D and BMI were consistent across the three groups (non-NAFLD; steatosis; fibrosis; Table 1). The high-BMI non-NAFLD liver control group may have helped to reduce the influence of obesity-related risk factors on the data. In other words, neither obesity nor T2D appeared to progress among the groups studied here. Additionally, T2D interventions (e.g., Metformin) potentially reduced metabolite variation [29], although more work is needed. Overall, obesity- and T2D-dependent metabolite trends are therefore expected to be reduced in this study, and it is reasonable that BCAA and AAA did not change significantly between the three groups in Table 2.

### 2.5. Decreasing BCAA as a Function of Fibrosis Stage

The fibrosis group was further divided into stage 1 (*n* = 16) and stages 2 and 3 (*n* = 13) subgroups. While BCAA were consistent among the three broader groups (Table 2), there is a decreasing trend in BCAA between stage 1 and stages 2 and 3 fibrosis samples (Table 4). Changing BCAA levels are commonly interpreted with respect to insulin resistance and T2D in obesity-related studies; however, the T2D rates and insulin levels here do not support these as possible factors (Table 4). A decrease in BCAA, as well as an increase in AAA, is associated with advanced liver disease, including hepato-cellular carcinoma (HCC) and is characterized as the BCAA/AAA ratio (Fischer ratio) [35,36]. The BCAA/Tyrosine Ratio (BTR) is also supported as a metric for the risk and advancement of liver disease [37], while decreased tyrosine is associated with poorer survival outcomes of HCC [38]. The decreasing BCAA observed here for fibrosis progression may therefore signal risk of progression to more advanced liver disease such as cirrhosis [35,36,37,39]; however, the potential for observing changes in AAA with fibrosis progression in Table 4 is less clear in this work. Alternately, decreasing BCAA has also been associated with loss of kidney function [40]. Note that decreasing NMR-derived serum creatinine is interpreted here to be dictated by loss of liver function between stage 1 and stages 2 and 3 fibrosis groups. The suppression of serum creatinine in liver fibrosis means that creatinine is difficult to interpret relative to kidney function [41]. To consider the possible role of kidney function, we examined the clinical determination of glomerular filtration rate (GFR), which shows no change with the progression of fibrosis in Table 4. While this work leaves open a hypothesis that BCAAs may be a potential signal of changing kidney health during the progression of fibrosis, the decreasing BCAAs observed here in Table 4 are more likely reporting on fibrosis progression and could signal risk of advanced liver disease. Although there are relatively low sample numbers within the fibrosis stages (Table 4), and these results should be interpreted cautiously, these key BCAA metabolites (isoleucine, leucine, valine, creatinine) all decreased with *p* < 0.005 and should be investigated further.

### 2.6. TCA Cycle and Urea Cycle Metabolites: Steatosis

**Figure 3:**
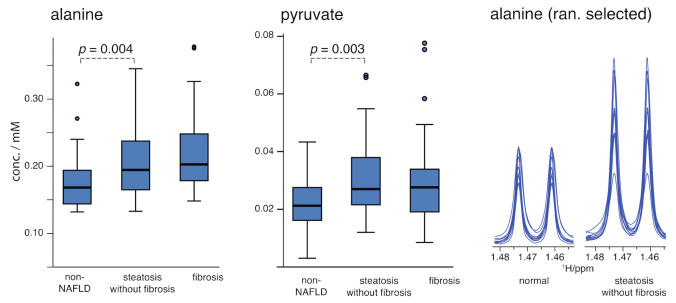
Boxplots of alanine and putative pyruvate concentrations are shown as a function of group membership. In the final panel, the spectral region corresponding to the doublet from the methyl sidechain is depicted for 10 randomly selected spectra in each of two groups to illustrate the practical spectral differences. Multiply concentrations by 2 for the serum levels.

Increasing alanine between the three groups is among the more significant findings (*p* = 0.003) in the comparison of the three broad groups (Figure 3), with a larger change occurring between the non-NAFLD liver and steatosis groups (*p* = 0.004). A putative pyruvate signal (Figure 3) also increases between the non-NAFLD and steatosis groups. Pyruvate is assigned from a singlet but occurs in a well-conserved location. A coincidental singlet from a novel metabolite that varies strongly with NAFLD and is consistent with increasing alanine cannot be ruled out but is unlikely.

**Figure 4:**
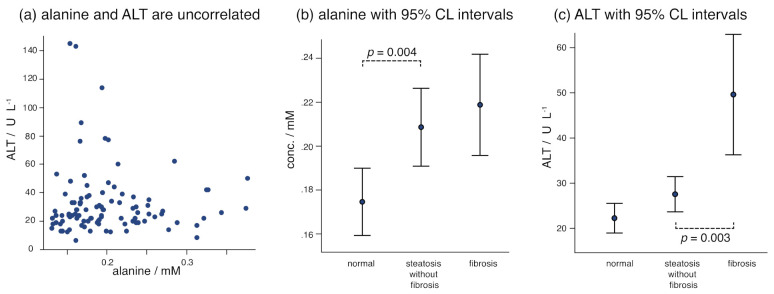
In this study, serum alanine increased particularly upon steatosis, whereas ALT increased more strongly in fibrosis. While energy (TCA cycle) disruption is supported by these data, trends here could also be influenced by urea cycle disruption in steatosis that builds up concentrations of alanine in steatosis, followed by overexpression of ALT in response to fibrosis.

Changes in alanine and pyruvate broadly indicate dysregulation of the alanine/glucose cycle (a.k.a. Cahill cycle) and can be interpreted to further implicate dysregulation of the TCA cycle. However, as alanine is the primary nitrogen shuttle in the body, increasing alanine, together with pyruvate, could also be consistent with dysregulation of the hepatic urea cycle, causing an accumulation of these key metabolites [42,43]. Furthermore, an interesting observation is that alanine and ALT are uncorrelated in this work (Figure 4), where alanine varies more strongly with steatosis but ALT varies more strongly in fibrosis. The lack of correlation (Pearson coefficient = −0.044, *p* = 0.663) suggests considering mechanisms in which they are independently modulated; a disruption of the urea cycle could be proposed to explain a buildup of alanine levels in steatosis, while overexpression of ALT in response to hepatic damage occurs later in fibrosis. Notably, the lack of correlation among two variables that are both sensitive to progression suggests their utility in classification modeling, which is presented in the discussion.

## 3. Discussion

Metabolic syndrome (MetS) is an umbrella of risk factors leading to increased incidences of serious conditions such as type 2 diabetes (T2D) and heart disease. A global health crisis, nonalcoholic fatty liver disease (NAFLD) represents the hepatic progression of MetS and, particularly in developed regions, approximately 25–30% of the population exhibits steatosis as the first stage of NAFLD [44], marked by excess fat deposits in the liver. About 10–20% of that group (i.e., 2–6% of the general population) advances to more serious stages of inflammation and fibrosis, which can further advance to cirrhosis and hepatocellular carcinoma (HCC) if severe or untreated.

There is an unmet need for improved characterization of the metabolic progression of NAFLD. Notably, the evolving study of NAFLD has led to a recommendation to associate hepatic steatosis with declining metabolic health and to diagnose it as metabolic associated fatty liver disease (MAFLD) [45]. Additionally, hepatic steatosis >5% is best detected with an invasive histological biopsy, but if predictors of metabolic dysregulation can be obtained from blood draws and multi-omics methods, then the detection of NAFLD presence, risk, and advancement would be transformed.

### 3.1. Biobanked Sera of Bariatric Surgery Patients

**Figure 5:**
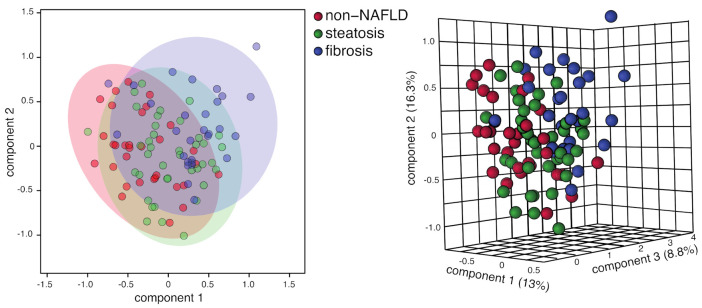
Log normalized PLS-DA clustering of all three groups (performed with Metaboanalyst [46]). Comparable results are similar for different normalizations schemes and also when excluding lactate and glucose. Significant variables (VIP > 1.2) are 2-hydroxyisovalete (3.5), acetone (3.2), 3-hydroxybutyrate (2.1), pyruvate (1.6), and azelate (1.3). Note that in the right-hand panel, relatively clear separation between fibrosis and non-NAFLD groups is demonstrated with PC1 and PC3 (i.e., the right panel shows a rotation around the PC2 axis relative to the projection in the left panel).

A broad finding is that the results in this work support the idea that retrospective, standard-of-care sera can be useful in discovering significantly varying metabolites in non-clinical populations composed of different states of NAFLD, advancing a larger hypothesis that metabolite trends are accessible in biobanked sera [7]. Significant trends were observed in a small number of key metabolites (Table 2 and Table 3), even though these samples can represent more variation in the pre-draw conditions (e.g., fasting, time of draw, diet and over-the-counter (OTC) drug monitoring, etc.) than in prospective clinical research studies. For example, some samples showed acetaminophen and caffeine; a small number of samples include pioglitazone use, and we do not exclude those either. Additionally, use of over-the-counter supplements such as vitamin E was not monitored and could occur. The intra-group variation is apparent, for example, even when applying supervised approaches such as partial least squares discriminant analysis (PLSDA), shown in Figure 5 and Figure S1. Despite the intra-group variation, the PLSDA analysis distinguishes the fibrosis group relatively well from the non-NAFLD liver group (Figure 5 and Figure S1), with the steatosis group scores occurring in between them (Figure 5). Histology notes were reviewed for one sample, classified as a non-NAFLD liver but which clustered well in the fibrosis PLSDA group, and for another sample, classified as steatosis but which clustered in the fibrosis PLSDA group. No evidence to update the assessments was found; however, the sample classified as steatosis was limited by heat artefacts and it cannot be ruled out that a different section might have yielded different information.

Prospective clinical metabolomics offers several advantages, such as controlling numerous variables and monitoring compliances in order to measure weak trends, obtain unique clinical data, isolate specific disease states, and respond to health crises in real time (e.g., SARS-CoV-19 metabolomics). However, as biobanks grow, the potential to design increasingly sophisticated studies from them will only increase, and this work supports the growth of retrospective metabolomics studies.

### 3.2. Overview of Markers for NAFLD

In these groups comprised of morbidly obese female patients, known clinical indicators of NAFLD (e.g., insulin, triglycerides, alanine aminotransferase, aspartate aminotransferase) changed significantly between the groups. Among the NMR-derived markers, inhibition of fatty acid oxidation was clearly observed in the steatosis group relative to the non-NAFLD liver group through increasing ketone bodies. The localization of increasing ketone bodies to the steatosis group in this study is noted; increasing ketone bodies also distinguished steatosis in HCV patients [34]. Increasing alanine and pyruvate (putative) are frequently associated with NAFLD progression, also in mouse models [47], and are localized to steatosis in this study, where alanine and pyruvate means increased by about 20% and 40% in the steatosis group relative to the control group. Neither BCAAs nor AAAs changed significantly with the progression of NAFLD between the three groups studied here, in which obesity and diabetes were consistent between the groups (Table 1).

The markers (Figure 3 and Figure 4) observed here to be associated with steatosis could implicate the TCA cycle broadly and support mitochondrial disruption as a feature of NAFLD, but they are also consistent with a growing hypothesis of hepatic urea cycle disruption in NAFLD [42,43,47]. The results of this work, particularly the strong alanine increase in the steatosis group, may help to localize urea cycle disruption to steatosis. Decreasing serum betaine is widely reported in NAFLD, and it is also confirmed here, where this work observed that serum betaine decreased significantly in the fibrosis group relative to the steatosis group. Additionally, betaine appeared to decrease weakly with fibrosis progression (*p* = 0.058, Figure S5, Supporting Information). Significant metabolites observed here in the steatosis group relative to a non-NAFLD liver are particularly similar to several significant metabolites in a study of NAFLD progression for patients with hepatitis C (e.g., ketone bodies, alanine, pyruvate, suberate) [34]. It is remarkable that, except for ALT, the panel of biomarkers distinguishing the steatosis and fibrosis groups is completely distinct from the panel distinguishing the non-NAFLD and steatosis groups (Table 3). In other words, this work supports a discrete shift in metabolism between steatosis and fibrosis. If TCA and urea cycles are dysregulated in steatosis, then the complex responses in dealing with hepatic damage represent a distinct metabolic direction in fibrosis. This finding should have utility in the development of exploratory classification models, explored below.

The interplay of BCAAs, diabetes, and hba1c should be considered further. As in this work, a recent study also did not see changes in serum BCAAs in the progression of NAFLD, where obesity and diabetes were also consistent across the groups considered [10]. An increased incidence of Type 2 Diabetes with NAFLD [48] means that biomarker discovery often must take place in studies in which T2D rates increase with NAFLD progression [9,13]. As BCAAs are well known to increase with the risk and development of diabetes [8,49,50,51], their changes could be attributed to T2D, NAFLD, or to an interaction between them. Increasing BCAAs have been noted in liver tissue in NAFLD progression [18,52], and comparing serum and tissue levels of BCAAs in NAFLD could help to better understand their trends in NAFLD. Given the consistent rates of obesity and diabetes between the groups in Table 1, which are also supported by BCAA/AAA levels not changing significantly, the increasing hba1c trend in this work is attributed to NAFLD progression and is consistent with the use of hba1c as a reporter on NAFLD [27]. 

There is a widely recognized need to develop noninvasive classifiers for NAFLD progression to cope with the clinical dilemma of identifying the small set of patients at risk for progression beyond steatosis. The potential to develop omics-based classifiers with high sensitivity/specificity scores has been demonstrated with both triglyceride panels in lipidomics [53] and a multi-omics classifier [54]. While the current data set does not permit discovery and validation classification modelling, it is sufficient to test the hypotheses that aqueous metabolites can be used in NAFLD classification in non-clinical groups and further that key clinical variables can be complementary to NMR-derived metabolomic knowledge to improve classification.

First, while intra-group variation in biobanked samples is expected to affect cluster analysis, meaningful separation was obtained with good agreement between important variables in PLSDA (Figure 5 and Figure S1) and their means which varied significantly across groups (Table 2 and Table 3). Next, exploratory classification by logistic regression was conducted on these variables, summarized in Table 5. Classification percentages on the order of 75-85% are obtained, where success can be consistently improved through productive combinations of metabolite and clinical variables. Attention is drawn to the combination of alanine and ALT, where these variables both report on NAFLD progression but are remarkably uncorrelated (Figure 4), an important characteristic of variables in regression models. For example, just the combination of alanine (NMR) with clinical ALT and AST levels led to about 70% overall classification between non-NAFLD and steatosis subjects, and one additional metabolite increased overall success to about 78% (Table 5).

The prospects for distinguishing fibrosis from steatosis with logistic regression models are slightly improved, where models using variables noted in the clustering and means analyses yield overall success rates of about 85%. The results in Table 5 show good classification performance with small numbers of NMR-derived aqueous metabolites that are consistent with NAFLD pathology, supporting that the classification is neither fortuitous nor a result of overfitting. The inclusion of key clinical variables to further strengthen the models suggests the potential to develop a nonsurgical multi-component classifier that incorporates aqueous metabolites into broader omics knowledge of NAFLD progression [53,54].

Some markers noted in this work (Table 3) as changing significantly upon steatosis relative to a non-NAFLD liver (e.g., 3-hydroxybutyrate) are associated with NASH in other work [13]. Possible reasons for these differing results are discussed further in the Supporting Information (Figure S6). However, as this work supports the idea that ketone bodies are depressed in steatosis and stay depressed for subsequent disease progression, it is consistent that ketone bodies did not change in recent work focusing on progression beyond steatosis [10].

This work used extracted sera. Native sera can be attractive to use but do require suppression of broad background signals, may develop losses from filtration, and can experience line broadening when metabolites interact with dissolved proteins and lipids. Comorbidities can also cause confounding variation. For example, high ketone bodies could signal the presence of ketoacidosis in T2D subjects. Indeed, a small number of subjects in the steatosis and fibrosis groups displayed elevated values of 3HB, acetoacetate, and acetone, suggestive of the presence of confounding ketoacidosis. Overall, the findings reported here independently confirm the importance of key serum metabolites associated with NAFLD by NMR, but in this work some of these trends are associated with steatosis in middle-aged, high BMI female groups. The possible role of changing diabetes rates, including ketoacidosis, appeared to be reduced in this work, but such effects should be carefully considered in future metabolomic NAFLD work.

Tryptophan has been associated with NAFLD in some studies [9,10,55,56], but it did not show any significant change in this work (*p* = 0.242). Although it is tempting to inspect the means in Table 2 to speculate on a possible increase in tryptophan (or the other AAAs tyrosine or histidine) in the progression of NAFLD, note that tryptophan may have decreased weakly as fibrosis advanced from stage 1 to stages 2 and 3 (*p* = 0.051, Table 4). These variables could be more carefully isolated in prospective, controlled clinical research studies, but might not show significance when studies contain more intra-group variation.

## 4. Materials and Methods

### 4.1. Organic Extraction of Aqueous Metabolites 

The extraction was initiated with 200 μL aliquots of frozen serum samples in 1.5 mL microcentrifuge tubes. Once the serum was thawed, 400 μL of acetonitrile was added and the sample vortexed for 1 min. Acetonitrile solubilizes aqueous metabolites, precipitates proteins, and strips viral membranes to promote subsequent safe sample handling. A number of solvent systems are employed for extracting metabolites from sera [32,57,58,59,60], where the use of acetonitrile yields high metabolite recoveries, and methanol protocols may offer even more recovery [32]. The 1:2 (serum:ACN) ratio employed here yields high levels of metabolites with a strong reduction in macromolecular constituents, and also was shown to have linear recovery and reproduce ground truth for selected metabolites in mock samples [60]. Samples were always on ice or refrigerated during handling, with one exception noted below. Each sample was re-vortexed for 30–45 s at 4 °C to ensure proper mixing and disruption of debris prior to centrifuging for 10 min at 4 °C and 10,600 rcf on a benchtop refrigerated centrifuge, after which it was often found that the supernatant consisted of two liquid phases, an intriguing behavior since these solvents are miscible in all proportions [24]. We have reported and resolved the phase separation separately [24]. Briefly, the two-phase region is attributed to a third component (lipids), and this region can be avoided by working briefly at room temperature. Samples were re-vortexed at room temperature for 30–45 s, rested for 1 min at room temperature, and centrifuged for 5 min at room temperature >15k RCF. This short corrective procedure yields a single phase without observable protein contaminants. Room temperature refers to a laboratory maintained at an average temperature of 293 K. The supernatant was transferred to a new microcentrifuge tube and subjected to a centrifugal evaporator (1 torr) for 4 h to remove solvent. Sample stability during centrifugal drying is attributed to evaporative cooling, oxygen deprivation, and the stability of the dried pellet. Dried samples were stored in a −80 °C freezer and resuspended at time-of-use in an NMR buffer (0.10 mM DSS, 99% D_2_O, 75 mM sodium phosphate, pH 7.4).

### 4.2. Serum Collection and Group Design

Standard-of care serum samples from bariatric surgery patients were collected into Serum Separation Tubes (SST) (BD Diagnostics, Franklin Lakes, NJ, USA) per the standard clinic procedure. Briefly, blood samples were drawn into SST, set in room temperature for 30–60 min for clotting, and centrifuged for 15 min at 1500 RCF. Sera were then aliquoted and banked in a −80 freezer. No visually hemolytic sera were present.

Informed consent was obtained from all subjects involved in the study. The serum samples and clinical data for this study were obtained through an IRB approved research study (IRB #2004-0255) that includes a registry and biobank of bariatric surgery patients from Geisinger Health System [61]. The study included patient informed consent and conforms with the Declaration of Helsinki Statement. As part of the bariatric research program, serum samples were collected approximately 2–5 months before primary bariatric surgery and stored in the biobank. In concurrence with the bariatric surgery, a liver wedge biopsy is obtained from a consistent anatomic location per clinical standard of care. The biopsies were fixed in neutral buffered formalin and stained with hematoxylin and eosin for histological evaluation of steatosis and fibrosis using NASH CRN criteria [62]. All liver biopsies were read by experienced pathologists. Steatosis was graded as minimal (<5% of parenchyma), mild (5–33%), moderate (34–66%), or severe (>66%). Fibrosis stage was recorded as 0 (none), 1a (mild perisinusoidal), 1b (moderate perisinusoidal), 1c (portal/periportal), 2 (perisinusoidal and portal/periportal), 3 (bridging), or 4 (cirrhosis). Patients selected for this study were a convenience sample with available serum with paired liver histology. A review of liver biopsy evaluation in the context of fatty liver disease is given by E. Brunt [63]. Patient sera had not been used for any prior research. Patients were candidates for bariatric surgery (BMI > 40 or BMI > 35 with a metabolic disease such as diabetes, hypertension, hyperlipidemia, sleep apnea), chose to undergo bariatric surgery, and completed bariatric surgery. As such the patients had to be able to safely undergo surgery and were not medically fragile, had no major psychiatric issues, no major eating disorders, and were able to quit smoking. Ethnicity and race were self-disclosed, and the subjects are from a rural, generally Caucasian, geographically large, central Pennsylvania (USA) demographic region. 

Metformin use could modulate T2D and associated metabolites, but was not examined since this is a study of retrospective biobanked standard of care sera, and compliance could not be determined. The majority of the T2D patients were treated with Metformin, while some without T2D may also be treated with Metformin such as for pre-diabetes, IR, or PCOS.

### 4.3. Targeted Profiling 

Spectra were imported in to Chenomx 8.1 (Chenomx Inc., Edmonton, AB, Canada) and treated with line broadening (0.2 Hz), phasing, baseline correction, and reference deconvolution prior to profiling the assignable metabolites. In a small number of cases, reference deconvolution was deemed unnecessary. A rubric jointly developed by DSR/MCT/EJR was employed, where we note that automated profiling was used for many metabolites. The 100 spectra were processed and profiled by one blinded rater (DSR); a blinded and independent analysis jointly conducted by EJR and MCT yielded comparable results. One missing value, a hypoxanthine level that could not be determined in one sample due to the presence of additional signals, occurs in this dataset. 

In total, 49 metabolites and, in some cases, several active ingredients (acetaminophen, salicylic acid, ibuprofen, caffeine), were profiled (Table 2). A putative dimethylamine (DMA) signal was observed in some spectra and could not be profiled under these conditions. Uridine was frequently observed, but was deemed below the limit of quantitation in the majority of spectra and was not considered in the analysis. Trimethylamine-n-oxide (TMAO) was suggested in a small number of spectra, but low levels and resolution constraints prohibited profiling TMAO. Acetoacetate was profiled in the presence of large confounding signals (particularly valine) and its levels are considered to be estimates. Valproic acid was not detected in any sample. Note that the limits of detection and quantitation in ^1^H-NMR metabolomics at this field strength (600 MHz) are typically of the order of a few micromolar. Concentrations in Table 2 are a factor of 2 smaller than their circulating serum values since metabolites were obtained from 200 μL of serum, but reconstituted to a final volume of 400 μL in NMR buffer. Acetaminophen was profiled in 10 subjects (3 non-NAFLD, 2 steatosis, 5 fibrosis), caffeine in 28 subjects (7 non-NAFLD, 9 steatosis, 12 fibrosis), salicylate acid in 5 subjects (2 non-NAFLD, 1 steatosis, 2 fibrosis), and ibuprofen in one subject (non-NAFLD). Note that other subjects’ sera could also have these compounds, but at levels below the NMR detection limit.

### 4.4. Statistical Methodology and Data Analysis 

Descriptive statistics (means, standard deviations, frequencies, proportions) were computed for patient characteristics and metabolite and clinical serum concentrations. Groups were compared on categorical clinical variables using exact chi-square tests. Continuous variables for groups of two were compared using independent-samples t-tests, and in cases of strong skewness or other strong non-normality features, Mann–Whitney U tests were used. Continuous variables for groups of three were compared using ANOVAs and the more robust Welch and Brown–Forsythe tests; in cases of strong skewness or other strong non-normality features, Kruskal–Wallis tests were used. Classification by means of binary logistic regression was explored to see if NAFLD groups could be separated using a few metabolites and/or clinical variables. IBM SPSS Statistics 25, 26, and 28 were used for all analyses except PLSDA (Metaboanalyst [46]). Extreme outliers occurred in only three distributions. The data for acetate, creatine, and dimethylsulfone each had one extreme outlier, and all were retained. For these cases, the distance between the outlier and the next largest value was more than 7 standard deviations. Samples were strictly deidentified prior to handling. Extractions, NMR spectroscopy, data processing, and metabolite profiling were blinded to group membership. All subjects were retained in data analysis. Prior to data analysis and during the blinded phase of the work, one subject was removed when an audit of the metadata showed they did not meet group criteria (sample was obtained from a revision surgery, not the initial bariatric surgery).

### 4.5. NMR Data Acquisition 

Presat-NOESY [64] one dimensional ^1^H-NMR spectra were acquired at 14.1 T (Varian DD1, 600 MHz, 298 K) using a room temperature inverse probe. There is an approx. 2 hr window from resuspension in which to measure spectra [60], so each sample was acquired for 1 hr (typical pw_90_ = 6.5 μs; typical DSS linewidth = 0.90 Hz; 256 scans; 8 steady state scans; 100 ms NOESY period); each scan consumed 15 s (4 s acquisition; 11 s recycle time). The presat period was 2 s (included in 11 s recycle time).

## 5. Conclusions

The potential for aqueous metabolites to report effectively on NAFLD progression in biobanked sera is demonstrated with a non-high-throughput NMR study of obese female bariatric surgery patients. Changing metabolite levels show fatty acid oxidation dysregulation in steatosis and are consistent with an emerging view of hepatic urea cycle dysregulation in steatosis. Tissue damage as well as decreasing liver function in fibrosis are reported by a set of metabolite markers distinct from those identified in the steatosis group and which are consistent with inflammation and loss of liver function. Ketone bodies are sensitive reporters of fatty acid oxidation, but the presence of ketoacidosis may complicate the interpretation of ketone bodies. This work suggests that BCAA changes are not observable when obesity and diabetes do not change across NAFLD groups. However, the data clearly detect decreasing BCAA in advanced stages of fibrosis that may signal risk of advanced liver disease, which should be investigated further.

Exploratory modeling supports strategic combinations of aqueous metabolites and clinical variables that may be useful in classification modeling, where an important combination of NMR-derived alanine and clinical ALT levels improved NAFLD group classification in this work. Finally, this work identifies key aqueous metabolites that support further investigation for their inclusion in multi-omics models of NAFLD.

## Figures and Tables

**Table 1:** Means and standard deviations, or frequencies and proportion percentages, for the characteristics of the three groups studied in this work are shown. Two significant digits are retained in the standard deviation (SD), and the means rounded to the decimal place dictated by the SD.

Parameter	Non-NAFLD	Steatosis	Fibrosis	*p* (ANOVA)
Total, *n*	32	39	29	
Sex	F (32)	F (39)	F (29)	
Age (years)	45 ± 11	45 ± 11	43.9 ± 8.9	0.832
Race and Ethnicity ^d^	Caucasian (31) Native American (1)	Caucasian (38) African American (1)	Caucasian (29)	
BMI (kg/m^2^)	48.7 ± 6.9	50.1 ± 7.4	51.9 ± 8.0	0.268
Waist Circumference (inches)	50.5 ± 4.4	52.8 ± 5.5	54.0 ± 5.1	0.021
(Waist/cm)	(128 ± 11)	(134 ± 14)	137 ± 13	
Hypertension	0.41 (*n* = 13)	0.41 (*n* = 16)	0.45 (*n* = 13)	0.935 ^a^
Type 2 Diabetes	0.41 (*n* = 13)	0.38 (*n* = 15)	0.48 (*n* = 14)	0.713 ^a^
Hyperlipidemia	0.41 (*n* = 13)	0.31 (*n* = 12)	0.41 (*n* = 12)	0.632 ^a^
Blood sugar (mg/dL)	98 ± 40	107 ± 55	122 ± 64	0.472 ^c^
Insulin (µU/mL)	16 ± 11	26 ± 17	29 ± 16	0.003 ^b^
HbA1c (%)	5.85 ± 0.65	6.3 ± 1.2	7.0 ± 1.8	0.005 ^b^
Triglycerides (mg/dL)	150 ± 70	175 ± 92	280 ± 370	0.074 ^c^
Cholesterol (mg/dL)	192 ± 37	192 ± 44	201 ± 60	0.686
High-Density Lipoproteins (mg/dL)	53 ± 15	50 ± 11	48.0 ± 9.7	0.276
Low-Density Lipoproteins (mg/dL)	110 ± 37	107 ± 43	108 ± 42	0.961
Alanine Aminotransferase (U/L)	22.3 ± 9.1	28 ± 12	50 ± 35	<0.001 ^b^
Aspartate Aminotransferase (U/L)	23.8 ± 8.3	23.9 ± 6.8	42 ± 30	0.001^b^
Lobular Inflammation (0/1/2/3)	32/0/0/0	30/8/1/0	2/17/10/0	
Ballooning (0/1/2)	32/0/0	30/5/4	4/13/12	
Steatosis Grading				
0 (<5%)	32 (100%)	0 (0%)	0 (0%)	
1 (5–33%)	0 (0%)	26 (67%)	0 (0%)	
2 (34–66%)	0 (0%)	12 (31%)	15 (52%)	
3 (>66%)	0 (0%)	1 (3%)	14 (48%)	
Fibrosis Staging				
0 (none)	32 (100%)	39 (100%)	0 (0%)	
1a (mild perisinusoidal)	0 (0%)	0 (0%)	11 (38%)	
1b (moderate perisinusoidal)	0 (0%)	0 (0%)	2 (7%)	
1c (portal/periportal)	0 (0%)	0 (0%)	3 (10%)	
2 (perisinusoidal and portal/periportal)	0 (0%)	0 (0%)	8 (28%)	
3 (bridging)	0 (0%)	0 (0%)	5 (17%)	
4 (cirrhosis)	0 (0%)	0 (0%)	0 (0%)	

^a^ Exact chi-square test is reported. ^b^ Brown–Forsythe test is reported (skewness). ^c^ Kruskal–Wallis test is reported (extreme outlier or skewness). ^d^ See Flanagin et al. [25]; a demographic statement is included in Methods.

**Table 2:** Mean metabolite values, standard deviations, and one-way ANOVA significance values for 49 metabolites measured by ^1^H NMR (600 MHz; Chenomx 8.1) in the extracted sera (mM) across all three groups are listed. Multiply values by 2 to obtain the serum concentration. Two digits are retained in the standard deviation (SD) and the means rounded to the final decimal place dictated by the SD. Standard deviations in parentheses apply to the last digits of the mean, i.e., 0.1234 (56) means 0.1234 ± 0.0056.

Metabolite	Non-NAFLD (*n* = 32)	Steatosis (*n* = 39)	Fibrosis (*n* = 29)	*p* (ANOVA)
2-Aminobutyrate	0.0136 (66)	0.0114 (42)	0.0113 (40)	0.208 ^a^
2-Hydroxybutyrate	0.033 (22)	0.026 (13)	0.033 (14)	0.158 ^b^
2-Hydroxyisovalerate	0.0018 (13)	0.0021 (13)	0.0040 (42)	0.005 ^b^
2-Oxoisocaproate	0.0173 (64)	0.0177 (46)	0.0191 (64)	0.462 ^b^
3-Hydroxybutyrate	0.14 (13)	0.076 (83)	0.082 (93)	0.066 ^c^
3-Hydroxyisobutyrate	0.0057 (39)	0.0048 (18)	0.0057 (20)	0.273 ^b^
2-Methyl-3-oxovalerate	0.0109 (45)	0.0117 (32)	0.0132 (47)	0.114 ^b^
Acetate	0.033 (17)	0.039 (65)	0.0303 (61)	0.569 ^c^
Acetoacetate ^#^	0.0087 (65)	0.0071 (52)	0.0071 (50)	0.428
Acetone	0.014 (13)	0.0078 (53)	0.0070 (60)	0.003 ^b^
Alanine	0.175 (43)	0.209 (55)	0.219 (61)	0.003
Asparagine	0.0195 (57)	0.0201 (59)	0.0181 (54)	0.356
Aspartate	0.0134 (61)	0.0136 (61)	0.0133 (52)	0.964
Azelate *	0.035 (24)	0.032 (16)	0.048 (42)	0.086 ^b^
Betaine	0.0198 (64)	0.0208 (67)	0.0167 (52)	0.024
Carnitine	0.0168 (41)	0.0187 (45)	0.0188 (65)	0.154 ^a^
Choline	0.0079 (24)	0.0083 (22)	0.0075 (22)	0.328 ^b^
Citrate	0.0186 (55)	0.0179 (55)	0.0199 (54)	0.308
Creatine	0.026 (37)	0.0161 (67)	0.0186 (97)	0.445 ^c^
Creatinine	0.027 (12)	0.025 (12)	0.023 (13)	0.575
Dimethylsulfone	0.05 (27)	0.0036 (58)	0.005 (13)	0.937 ^c^
Formate	0.0231 (39)	0.0236 (34)	0.0222 (30)	0.258
Glucose	1.88 (55)	2.14 (75)	2.4 (1.3)	0.069 ^b^
Glutamate	0.045 (17)	0.050 (16)	0.054 (21)	0.180
Glutamine	0.209 (41)	0.207 (39)	0.195 (50)	0.410
Glycerol	0.049 (23)	0.050 (18)	0.058 (25)	0.163
Glycine	0.131 (39)	0.126 (27)	0.126 (43)	0.813 ^a^
Histidine	0.0344 (56)	0.0355 (59)	0.0344 (71)	0.696 ^b^
Hypoxanthine **	0.0059 (25)	0.0060 (27)	0.0042 (18)	0.006 ^b^
Isobutyrate	0.0056 (15)	0.0053 (14)	0.0057 (13)	0.530
Isoleucine	0.037 (18)	0.0365 (70)	0.0378 (86)	0.900 ^b^
Lactate	1.11 (43)	1.25 (38)	1.30 (40)	0.171 ^b^
Leucine	0.072 (30)	0.067 (13)	0.069 (15)	0.641 ^b^
Lysine	0.053 (14)	0.057 (11)	0.058 (13)	0.259
Mannose	0.0312 (61)	0.033 (12)	0.034 (12)	0.565 ^b^
Methionine	0.0135 (38)	0.0137 (28)	0.0132 (26)	0.821
Ornithine	0.026 (11)	0.0260 (68)	0.0244 (71)	0.730 ^b^
Phenylalanine	0.047 (14)	0.047 (12)	0.047 (10)	0.985
Proline	0.089 (36)	0.098 (33)	0.096 (40)	0.534 ^b^
Propylene glycol	0.088 (35)	0.092 (29)	0.117 (70)	0.050 ^b^
Pyroglutamate	0.0099 (45)	0.0108 (39)	0.0123 (54)	0.115
Pyruvate	0.0221 (93)	0.031 (14)	0.031 (17)	0.015 ^b^
Serine	0.045 (12)	0.046 (11)	0.045 (13)	0.924
Suberate *	0.035 (11)	0.035 (10)	0.042 (15)	0.087^a^
Taurine	0.074 (40)	0.070 (17)	0.056 (13)	0.031 ^b^
Threonine	0.049 (16)	0.053 (12)	0.048 (14)	0.232
Tryptophan	0.0254 (72)	0.0270 (63)	0.0281 (50)	0.242 ^b^
Tyrosine	0.040 (13)	0.0428 (91)	0.044 (12)	0.555 ^b^
Valine	0.117 (38)	0.118 (21)	0.123 (26)	0.689 ^b^

^a^ Levene’s test for homogeneity of variances is significant (*p* < 0.05) and Welch’s test is reported. ^b^ Brown–Forsythe test is reported (skewness); ^c^ Kruskal–Wallis test is reported (extreme outlier or skewness). ^#^ due to overlap with stronger signals, acetoacetate concentrations are estimates and significance may be unreliable. * suberate is considered to be confounded with pimelate; azelate is considered confounded with sebacate. ** one missing hypoxanthine value in the non-NAFLD liver group due to confounding signals.

**Table 3:** Independent-samples *t*-tests among the three groups (Table 1).

Non-NAFLD vs. Steatosis		Steatosis vs. Fibrosis		Non-NAFLD vs. Fibrosis	
Metabolite (NMR)	*p*	Metabolite (NMR)	*p*	Metabolite (NMR)	*p*
3-hydroxybutyrate *	0.035	2-Hydroxybutyrate	0.045	2-Hydroxyisovalerate	0.010
Alanine	0.004	2-Hydroxyisovalerate	0.023	Acetone	0.007
Acetone	0.012	3-Hydroxyisobutyrate	0.046	Alanine	0.002
Pyruvate	0.003	Betaine	0.008	Betaine	0.041
		Hypoxanthine	0.003	Hypoxanthine ^#^	0.006
		Suberate	0.031	Taurine	0.025
		Taurine	0.001		
		2-Hydroxybutyrate	0.045		
Clinical	*p*	Clinical	*p*	Clinical	*p*
Waist	0.049	ALT	0.003	Waist	0.005
Insulin	0.006	AST	0.003	Insulin	0.001
HbA1c	0.046			HbA1c	0.002
ALT	0.042			Triglycerides *	0.029
				ALT	<0.001
				AST	0.004

^#^ one missing value of hypoxanthine in the non-NAFLD group; * Mann–Whitney U test cited.

**Table 4:** Comparison of metabolite means as a function of fibrosis staging, with selected clinical variables. *p* values obtained from independent-samples *t*-test. Multiply concentrations by 2 for the original serum levels.

	Fibrosis Stage 1 (*n* = 16)	Fibrosis Stages 2 and 3 (*n* = 13)	*p* Value
Metabolite (NMR)			
2-Aminobutyrate	0.0128 (41)	0.0094 (32)	0.021
Acetoacetate	0.0087 (57)	0.0053 (33)	0.036 ^#^
Creatinine	0.0295 (98)	0.016 (13)	0.001 ^#^
Hypoxanthine	0.0051 (16)	0.0032 (15)	0.003
Isoleucine	0.0422 (72)	0.0324 (71)	0.001 ^#^
Leucine	0.076 (12)	0.059 (14)	0.001
Lysine	0.063 (12)	0.052 (11)	0.020
Methionine	0.0141 (26)	0.0122 (21)	0.040
Tyrosine *	0.048 (13)	0.0385 (85)	0.050 ^#^
Valine	0.135 (21)	0.109 (24)	0.004
Clinical			
Type 2 diabetes	0.50 (8/16)	0.46 (6/13)	1.000 ^§^
Insulin	31 (15)	28 (17)	0.475 ^#^
HbA1c	6.5 (1.2)	7.6 (2.3)	0.232 ^#^
GFR ^+^	86 (20)	90 (23)	0.612

^#^ Mann-Whitney U test exact significance. ^§^ Exact chi-square test. * additionally, tryptophan (decreasing; *p* = 0.051) is weakly suggested. ^+^ glomerular filtration rate via Modification of Diet in Renal Disease (MDRD) Study method (mL/min/1.73m^2^).

**Table 5:** Exploratory logistic regression modeling shows that small numbers of NMR-derived aqueous metabolites are sensitive to NAFLD progression in non-clinical groups, and further that a complementary interaction of clinical variables is obtained. A particularly productive combination of alanine and ALT (alanine aminotransferase) is observed in this work.

Variables	Non-NAFLD and Fibrosis (% Classification Success)			
	Overall	Non-NAFLD	Fibrosis	Nagelkirke R^2^
Metabolite(NMR) only:				
alanine, acetone, betaine, 2hiv	85.2	90.6	79.3	0.478
betaine, hypoxanthine, tryptophan, taurine	80.0	83.9	75.9	0.590
Metabolite(NMR) + clinical:				
ALT, alanine, acetone, 2hiv	86.9	87.5	86.2	0.732
ALT, insulin, propylene glycol, 2hiv	86.2	89.7	82.8	0.726
	**Steatosis and Fibrosis (% Classification Success)**			
	Overall	Steatosis	Fibrosis	Nagelkirke R^2^
Metabolite(NMR) only:				
pyroglutamate, betaine, taurine, 2hb	85.3	89.7	79.3	0.507
azelate, hypoxanthine, taurine, 2hiv	82.4	87.2	75.9	0.471
Metabolite(NMR) + clinical:				
AST, taurine, azelate, 2hiv	85.3	89.7	79.3	0.587
AST, taurine, glucose, 2hiv	86.8	94.9	75.9	0.527
	**Non-NAFLD and Steatosis (% Classification Success)**			
	Overall	Non-NAFLD	Steatosis	Nagelkirke R^2^
Metabolite(NMR) only:				
acetone, alanine, pyruvate, creatine	73.2	65.6	79.5	0.314
acetone, alanine, pyruvate	70.4	65.6	74.4	0.272
Metabolite(NMR) + clinical:				
ALT, alanine, acetone, pyruvate	76.1	68.8	82.1	0.358
AST, ALT, alanine	78.6	77.4	79.5	0.305

2hiv = 2-hydroxyisovalerate; 2hb = 2-hydroxybutyrate.

## Data Availability

The data presented in this study are available on request from the corresponding author, because of its usage in the ongoing study.

## Supplementary Materials

The following are available online at https://www.mdpi.com/article/10.3390/metabo11110737/s1. Figure S1: Supervised clustering, Figure S2: Examining Acetoacetate, Figures S3 and S4: Weak trends suggesting liver damage and loss of liver function in fibrosis, Figure S5: Betaine and fibrosis staging, Figure S6: Comparison to prior work, Table S6: Comparison of selected data in this work to Mannisto et al.
