# Supplementary material for: Aqueous Metabolite Trends for the Progression of Nonalcoholic Fatty Liver Disease in Female Bariatric Surgery Patients by Targeted 1H-NMR Metabolomics
Source: Metabolites. 2021 Oct 27;11(11):737. doi: 10.3390/metabo11110737 (PMC8619318; doi:10.3390/metabo11110737)
Supplement: Supplementary file 1 [file metabolites-11-00737-s001.zip › metabolites-1398438-supplementary.pdf]

Supplemental Information for:

Aqueous metabolite trends for the progression of non-alcoholic fatty liver disease in female bariatric surgery patients by targeted ^{1}H-NMR metabolomics

Emma J. Robinson, Matthew C. Taddeo, Xin Chu[#], Weixing Shi[#], Craig Wood[#], Christopher Still[#], Virginia G. Rovnyak*, David Rovnyak

Bucknell University, Department of Chemistry, 1 Dent Drive, Lewisburg, PA 17837-2005

*University of Virginia School of Nursing, Charlottesville, VA

[#]The Obesity Institute, Geisinger, Danville, PA 17822

Contents:

S.1 : Supervised clustering
S.2: Examining Acetoacetate
S.3-S.4: Weak trends suggesting liver damage and loss of liver function in fibrosis.
S.5: Betaine and fibrosis staging.
S.6: Comparison to a prior study

Section S.1 : Supervised clustering

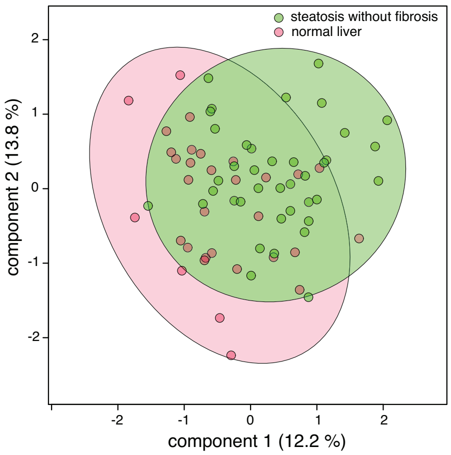

Variable Importance

metabolite	C1
3Hydroxybutyrate	2.9863
Pyruvate	2.5271
Acetone	2.3074
Alanine	1.8425
2Hydroxyisovalerate	1.7671
Acetoacetate	1.5315
Creatine	1.4821

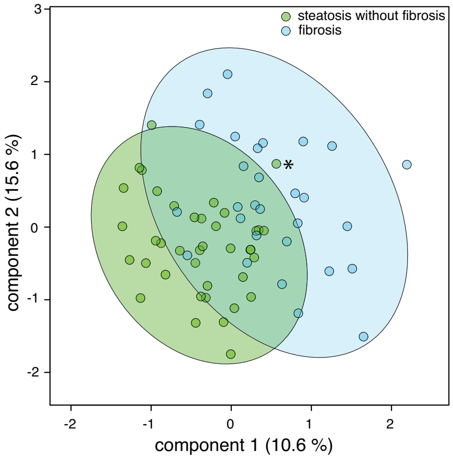

Hypoxanthine	2.5836
2Hydroxyisovalerate	2.2144
Taurine	1.9966
Betaine	1.9272
2Hydroxybutyrate	1.7908
Azelate	1.7749
Acetone	1.465
3Hydroxyisobutyrate	1.4499
Suberate	1.4152
PropyleneGlycol	1.2344
Glycerol	1.2036

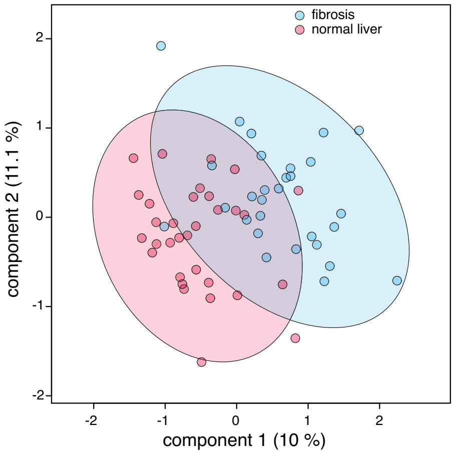

2Hydroxyisovalerate	2.7695
Acetone	2.5904
Pyruvate	1.7619
3Hydroxybutyrate	1.7052
Alanine	1.6903
3Methyl2oxovalerate	1.4026
Pyroglutamate	1.3741
PropyleneGlycol	1.291
Azelate	1.2272
Taurine	1.2261

Figure S.1 PLSDA results comparing groups indicates promising separation in all cases, although it can be seen that distinguishing steatosis from a normal liver is more challenging. (analyses performed in Metaboanalyst [1]). Data were log and pareto scaled, however similar results were obtained for different normalization schemes. The histology of one sample assessed to steatosis (asterisk*) but which clustered similarly with fibrosis samples was reviewed, and it was noted that this assessment was restricted by the presence of thermal distortion.

Section S.2: Examining Acetoacetate

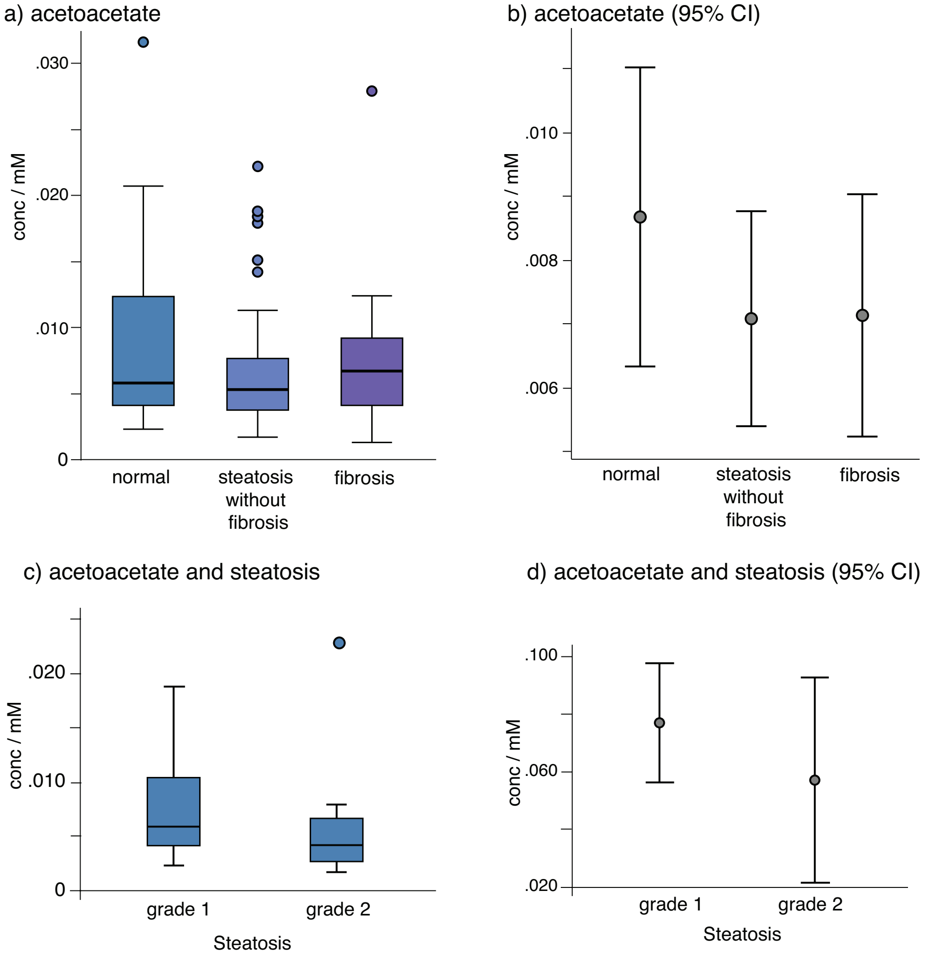

Figure S.2 Acetoacetate is difficult to profile due to spectral crowding in these data, but an attempt was made to profile it during the blinded phase of the work, given its importance to understanding ketone bodies in NAFLD. While the means decreased from normal to steatosis and fibrosis (**Table 2**, main body), the medians did not trend similarly as seen in panel (a) above, with the median for fibrosis even being the largest of the three groups. The ANOVA over the three groups was not significant ($p = .428$) either. These data allow for the possibility of a decreasing trend from normal to steatosis, but do not establish it. The steatosis group was examined further by comparing the grade 1 and grade 2 samples (panels c-d). Within the steatosis group of 39, 26 had grade 1 steatosis, 12 had grade 2, and one sample had grade 3. The means and medians both drop from grade 1 to grade 2, and, ignoring the one grade 3 case, the Mann-Whitney U test may indicate a drop in acetoacetate from steatosis grade 1 to grade 2 ($p = .106$). Concentrations of extracted samples reported (multiply by two for circulating serum level).

S.3-S.4: Weaker trends suggest liver damage and loss of liver function in fibrosis.

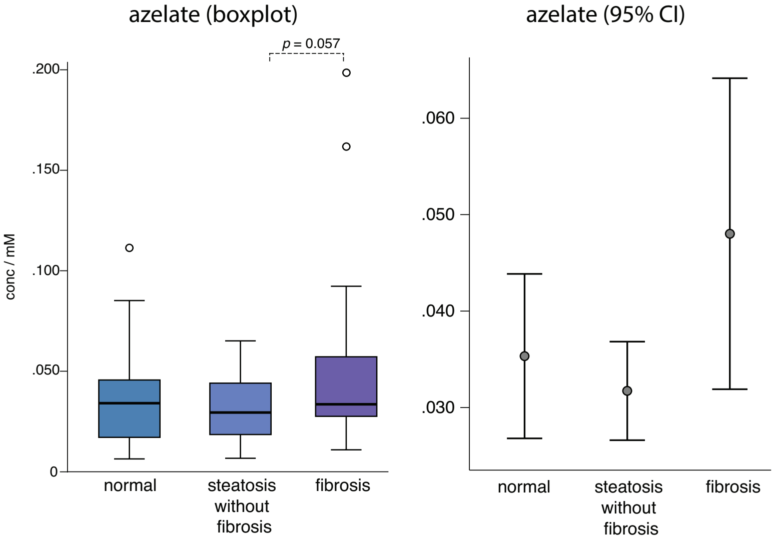

Figure S.3 A weakly increasing trend in the azelate concentrations in the fibrosis group may be useful given the significant increase in suberate in the fibrosis group (**Figure 2** of the main text); these data support that the medium chain fatty acids suberate, pimelate, azelate, and sebacate accumulate in circulating serum in the fibrosis groups and could reflect liver damage and a corresponding loss of liver function in fibrosis. As noted in the main text, azelate is expected to be confounded with sebacate, and suberate with pimelate. Multiply concentrations by two for circulating serum level.

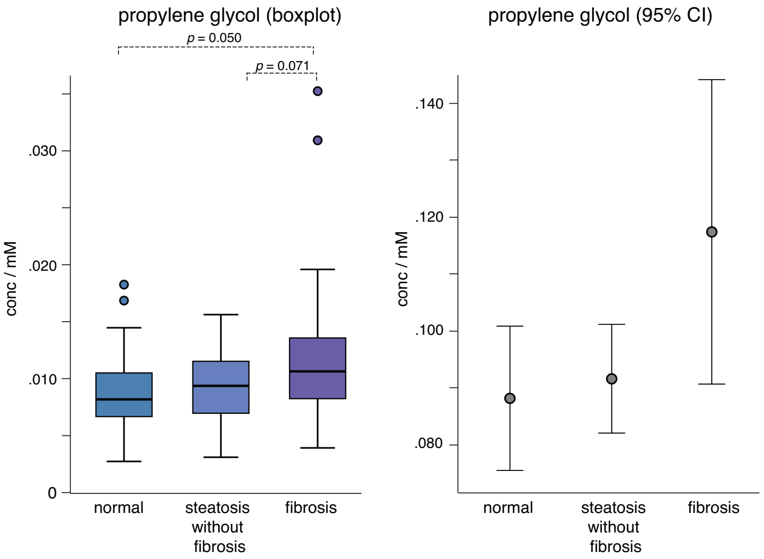

Figure S.4 Accumulation of propylene glycol is weakly suggested in NAFLD progression, consistent with reduced liver function in fibrosis. Multiply concentrations by two for circulating serum levels.

Section S.5: Betaine and fibrosis staging.

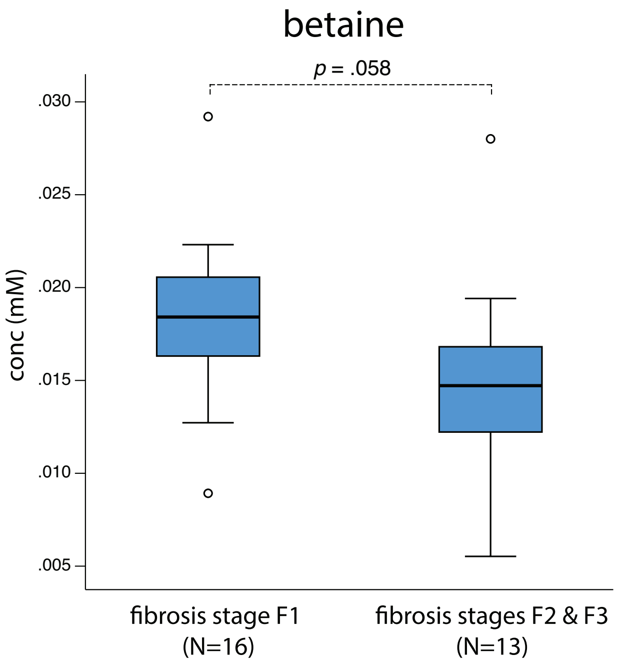

Figure S.5 Serum betaine may decrease in fibrosis progression in this work, where the determination of this trend is limited by the relatively small sample numbers. Multiply concentrations by two for circulating serum levels.

Section S.6: Comparison to a prior study

There are some similarities between this study and a prior NMR-based study by Mannisto et al., [2] summarized in **Table S.1** below. However the results reported here find decreasing ketone bodies in steatosis whereas the prior study observed ketone bodies decreasing in NASH.

Some differences are that this study was female only, had a larger steatosis group, used extracted sera, had slightly higher BMI averages, and used a non-high-throughput approach. Also, in the prior work [2], diabetes (T2D) increased significantly across the groups, where BCAA/AAA were also observed to increase. In this work, diabetes did not increase across groups (**Table 1** of main text) and BCAA/AAA did not change significantly, although intra-group variation may have masked weak trends in this work.

In the work reported here, the ketone body 3-HB (3-hydroxybutyrate, **Figure 1** in main text) decreased in the steatosis group relative to a normal liver and remained depressed in the fibrosis group. The data herein did not permit precise quantitation of acetoacetate (**Figure S.2**). In the prior work [2], 3-HB and acetoacetate increased noticeably in the steatosis group (significance not reported) and decreased in NASH (**Table S.1**). The increase they observed in ketone bodies in the steatosis group[2] could be due to ketoacidosis in some patients. For example, as noted in the main text, in this work a very small number of patients in the steatosis and fibrosis groups (ca. 2-3 in each group) exhibited strongly elevated ketone bodies suggestive of ketoacidosis, but the overall group sizes moderated their effect.

	**High BMI, Normal Liver**		**Steatosis**		**NASH or Fibrosis**	
Source	*Ref [2]*	*this work*	*Ref [2]*	*this work*	*Ref [2]* (NASH)	*this work* (Fibrosis)
**Characteristics**						
Sample Size	32	32	19	39	25	29
Age	47.9 ± 9.7	45 ± 11	45.8 ± 9.8	45 ± 11	46.7 ± 8.0	43.8 ± 8.9
Sex	11M/21F	32F	4M/15F	39F	10M/15F	29F
T2D	0.25 (*n*=8)	0.41 (*n*=13)	0.32 (*n*=6)	0.38 (*n*=15)	0.44 (*n*=11)	0.48 (*n*=14)
BMI	44.1 ± 6.8	48.7 ± 6.9	44.8 ± 4.3	50.1 ± 7.4	44.3 ± 6.9	51.9 ± 8.0
HDL (mg/dL)	41 ± 12	53 ± 15	39 ± 7	50 ± 11	41 ± 15	48.0 ± 9.7
LDL (mg / dL)	96 ± 27	110 ± 37	82 ± 31	107 ± 43	112 ± 39	108 ± 42
Triglycerides	132 ± 62	150 ± 70	129 ± 53	175 ± 92	154 ± 53	280 ± 370
**Selected Metabolites**						
Alanine	0.409 (60)	0.349 (85)	0.415 (50)	0.417 (109)	0.452 (70)	0.437 (121)
3-HB	0.237 (190)	0.270 (256)	0.388 (300)	0.151 (165)	0.169 (12)	0.164 (186)
Acetoacetate#	0.062 (40)	0.017 (13)	0.106 (90)	0.014 (10)	0.043 (20)	0.014 (10)
Glucose	5.5 (1.0)	3.8 (1.1)	5.4 (1.0)	4.3 (1.5)	5.9 (1.5)	4.9 (2.6)
Isoleucine	0.056 (10)	0.075 (36)	0.061 (10)	0.073 (14)	0.067 (10)	0.076 (17)
Leucine	0.081 (10)	0.144 (60)	0.087 (20)	0.135 (26)	0.099 (20)	0.137 (30)
Valine	0.217 (30)	0.234 (76)	0.221 (40)	0.237 (41)	0.24 (4)	0.246 (52)
Tyrosine	0.060 (10)	0.081 (25)	0.058 (10)	0.086 (18)	0.071 (10)	0.087 (24)

Table S.1 Comparison of selected data in this work to Mannisto et al. [2] For the columns which report 'This Work', the values in **Table 2** of the main body are doubled in order to obtain the concentrations in the original serum and facilitate a direct comparison. #See **Figure S.2** for further discussion of acetoacetate.

1. Chong, J.; Soufan, O.; Li, C.; Caraus, I.; Li, S.; Bourque, G.; Wishart, D. S.; Xia, J. MetaboAnalyst 4.0: towards more transparent and integrative metabolomics analysis. *Nucleic Acids Res*. **2018**.

2. Mannisto, V. T.; Simonen, M.; Hyysalo, J.; Soininen, P.; Kangas, A. J.; Kaminska, D.; Matte, A. K.; Venesmaa, S.; Kakela, P.; Karja, V.; Arola, J.; Gylling, H.; Cederberg, H.; Kuusisto, J.; Laakso, M.; Yki-Jarvinen, H.; Ala-Korpela, M.; Pihlajamaki, J. Ketone body production is differentially altered in steatosis and non-alcoholic steatohepatitis in obese humans. *Liver Int*. **2015**, *35*, 1853-1861.
